# A metagenomic study of methanotrophic microorganisms in Coal Oil Point seep sediments

**DOI:** 10.1186/1471-2180-11-221

**Published:** 2011-10-04

**Authors:** Othilde Elise Håvelsrud, Thomas HA Haverkamp, Tom Kristensen, Kjetill S Jakobsen, Anne Gunn Rike

**Affiliations:** 1 Norwegian Geotechnical Institute, Sognsveien 72, P.O. Box 3930 Ullevål Stadion, N-0806 Oslo, Norway; 2 Department of Molecular Biosciences, University of Oslo, Blindernveien 31, P.O. Box 1041 Blindern, N-0316 Oslo, Norway; 3 Microbial Evolution Research Group, MERG, Department of Biology, University of Oslo, Blindernveien 31, P.O. Box 1066 Blindern, N-0316 Oslo, Norway; 4 Centre for Evolutionary and Ecological Synthesis (CEES), Department of Biology, University of Oslo, Blindernveien 31, P.O. Box 1066 Blindern, N-0316 Oslo, Norway

## Abstract

**Background:**

Methane oxidizing prokaryotes in marine sediments are believed to function as a methane filter reducing the oceanic contribution to the global methane emission. In the anoxic parts of the sediments, oxidation of methane is accomplished by anaerobic methanotrophic archaea (ANME) living in syntrophy with sulphate reducing bacteria. This anaerobic oxidation of methane is assumed to be a coupling of reversed methanogenesis and dissimilatory sulphate reduction. Where oxygen is available aerobic methanotrophs take part in methane oxidation. In this study, we used metagenomics to characterize the taxonomic and metabolic potential for methane oxidation at the Tonya seep in the Coal Oil Point area, California. Two metagenomes from different sediment depth horizons (0-4 cm and 10-15 cm below sea floor) were sequenced by 454 technology. The metagenomes were analysed to characterize the distribution of aerobic and anaerobic methanotrophic taxa at the two sediment depths. To gain insight into the metabolic potential the metagenomes were searched for marker genes associated with methane oxidation.

**Results:**

Blast searches followed by taxonomic binning in MEGAN revealed aerobic methanotrophs of the genus *Methylococcus *to be overrepresented in the 0-4 cm metagenome compared to the 10-15 cm metagenome. In the 10-15 cm metagenome, ANME of the ANME-1 clade, were identified as the most abundant methanotrophic taxon with 8.6% of the reads. Searches for particulate methane monooxygenase (*pmo*A) and methyl-coenzyme M reductase (*mcr*A), marker genes for aerobic and anaerobic oxidation of methane respectively, identified *pmo*A in the 0-4 cm metagenome as *Methylococcaceae *related. The *mcr*A reads from the 10-15 cm horizon were all classified as originating from the ANME-1 clade.

**Conclusions:**

Most of the taxa detected were present in both metagenomes and differences in community structure and corresponding metabolic potential between the two samples were mainly due to abundance differences.

The results suggests that the Tonya Seep sediment is a robust methane filter, where taxa presently dominating this process could be replaced by less abundant methanotrophic taxa in case of changed environmental conditions.

## Background

The Coal Oil Point seep area (COP), located in the Santa Barbara Channel, California, is one of the most active seep areas in the world [1]. Seepage of the greenhouse gas methane and other hydrocarbons has occurred in this area for over 500 000 years [2]. The methane emitted from the COP is mainly of thermogenic origin and the daily emission has been estimated to be at least 40 metric tons [1,3].

At a global scale, the oceans only make up about 2% of the global methane emission budget [4]. This low level is explained by prokaryotic oxidation of methane in marine sediments and bedrocks before it reaches the water column [5].

The oxygen penetration level in marine sediments is shallow, so most of the methane oxidation takes place at anaerobic conditions. Anaerobic oxidation of methane (AOM) is assumed to be a coupling of reversed methanogenesis and sulphate reduction. This process is likely performed by the yet uncultured anaerobic methanotrophic archaea (ANME) in syntrophy with sulphate reducing bacteria (SRB). Based on phylogeny, ANME can be divided into three clades: ANME-1, ANME-2 and ANME-3 [6-9]. ANME-2 and ANME-3 are affiliated to the *Methanosarcinales*, while ANME-1 is only distantly related to the *Methanosarcinales *and *Methanomicrobiales *[7-9]. Both ANME-1 and ANME-2 are associated with sulphur reducing deltaproteobacteria of the *Desulfosarcina*/*Desulfococcus*-branch [7,9,10]. ANME-3 is mainly associated with SRB strains closely related to *Desulfobulbus *[6].

The reversed methanogenesis model for AOM has gained support by a metagenomic study on ANME at Eel River [11] and sequencing of an ANME-1 draft genome [12]. In these studies sequence homologues of all enzymes needed for CO_2_-based methanogenesis with exception of N^5^, N^10^-methylene-tetrahydromethanopterin reductase (*mer*) were identified. Methyl-coenzyme M reductase (*mcr*A) is assumed to catalyze the first step of AOM and the last step of methanogenesis, and is therefore a marker gene for both processes. Similarly, dissimilatory sulphite reductase (*dsr*AB) is often used as a marker gene for SRB [13].

When oxygen is present, aerobic methanotrophs are active in methane oxidation. Known aerobic methanotrophs include representatives of *Gammaproteobacteria*, *Alphaproteobacteria *and *Verrucomicrobia *[14-18]. These organisms convert methane to methanol using the enzyme methane monooxygenase [17]. The particulate, membrane bound version of methane monooxygenase (*pmo*A), found in all aerobic methanotrophs (with exception of *Methanocella*), is used as a marker gene for aerobic oxidation of methane [19]. The methanol formed is converted to formaldehyde, which is assimilated by one of two known pathways. Type I and type II methanotrophs utilize the ribulose monophosphate pathway and the serine pathway respectively. Type × methanotrophs use primarily the ribulose monophosphate pathway, but possess the enzymes needed for the serine pathway as well [20].

Stable isotope probing and sequencing of 16S rDNA and *pmo*A, as well as lipid biomarker analysis, have detected type-I aerobic methanotrophs in sediments and biofilms at the COP Shane and Brian seeps [21,22]. Recently, measurements of average δ^13^C of carbonates and lipid biomarkers associated with ANME and SRB also indicated occurrence of AOM at the Brian seep [23]. Another survey at the Brian seep detected ANME-2 at 6-9 cm bsf (below sea floor) by FISH (Fluorescent in situ hybridization) [24].

In the present study, we have used metagenomics to characterize the taxonomic and metabolic potential for both aerobic and anaerobic methane oxidation in two sediment samples from different depths at the Tonya seep (COP). By avoiding PCR amplification and primer target specificity, the metagenomics approach offered further insight into the taxonomy and metabolic potential of the prokaryotic communities of the methane seep sediments.

## Results

### Gas measurements and methane oxidation rate

The average methane oxidation rate based on 11 measurements in the top 15 cm of the seep sediments was 156 ± 64 nmol cm^-3 ^day^-1^. Still, the gas emitted from the Tonya seep sediments into the water phase contained a large fraction of methane. Even after travelling 25 m through the water column, where dissolved O_2 _and N_2 _entered the bubbles, the two gas samples contained 80.4% (gas sample I) and 68.1% (gas sample II) methane. When O_2 _and N_2 _were excluded, and the hydrocarbon and CO_2 _content were normalized, methane accounted for 93.6% in both gas samples. The remainder consisted of CO_2 _and short chain hydrocarbons (C2, C3, i-C4 and n-C4).

### Metagenome creation through filtering of reads

**Figure 1:**
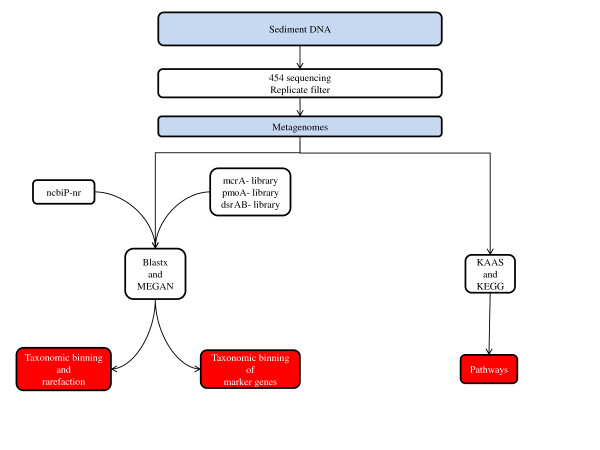
**Flowchart showing the workflow for taxonomic binning, marker gene annotation and pathway mapping**. Abbreviations used in the figure: ncbiP-nr (NCBIs non-redundant Protein Database), *mcr*A (methyl-coenzyme M reductase), *pmo*A (particulate methane monooxygenase), *dsr*AB (dissimilatory sulphite reductase), KAAS (KEGG Automatic Annotation Server) and KEGG (Kyoto Encyclopedia of Genes and Genomes).

454 sequencing resulted in 395540 reads for the 0-4 cm sample and 282964 reads for the 10-15 cm sample. Replicate filtering of the metagenomes removed 33.03% of the reads from the 0-4 cm sample and 31.31% of the reads in the 10-15 cm sample. The resulting metagenomes consisted of 264902 reads (average length 413 ± 138 bases, range 29-1907 bases) for the 0-4 cm sample and 194360 reads (average length of 419 ± 134 bases, range 29-1458 bases) for the 10-15 cm sample. All further analyses were performed on these metagenomes (Figure 1). Unless other ways specified, all percentages throughout the text are given as percent of total reads for each filtered metagenome.

Estimated effective genome sizes (EGS) were 4.8 Mbp and 4.0 Mbp for the 0-4 cm and 10-15 cm sample respectively (Additional file 1, Table S1).

### Rarefaction analysis

**Figure 2:**
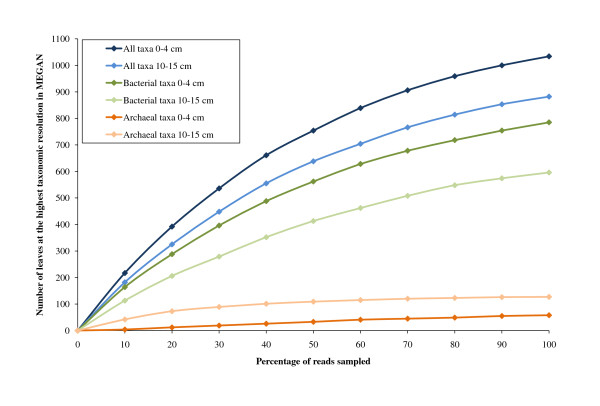
**Rarefaction curves created in MEGAN**. Rarefaction analysis was performed at the most resolved taxonomic level of the NCBI taxonomy in MEGAN for each metagenome. The curves for all taxa include *Bacteria*, *Archaea*, *Eukaryota*, Viruses, unclassified and other sequences.

Rarefaction analysis at the most resolved level of the NCBI taxonomy in MEGAN showed the taxonomic richness detected in the sediment samples (Figure 2). Including all assigned taxa, 1034 and 882 leaves were detected in the 0-4 cm and 10-15 cm metagenome respectively. Of these, 785 (0-4 cm) and 596 (10-15 cm) were bacterial and 58 (0-4 cm) and 127 (10-15 cm) archaeal. The rarefaction curves for bacterial and total taxa indicated that not all the taxonomic richness in the sediment was accounted for in our metagenomes. Still, the curves were levelling off from a straight line already at 10% of the metagenome size indicating repeated sampling of the same taxon. It is therefore likely that abundant taxa in the sediments were accounted for in the two metagenomes.

**Figure 3:**
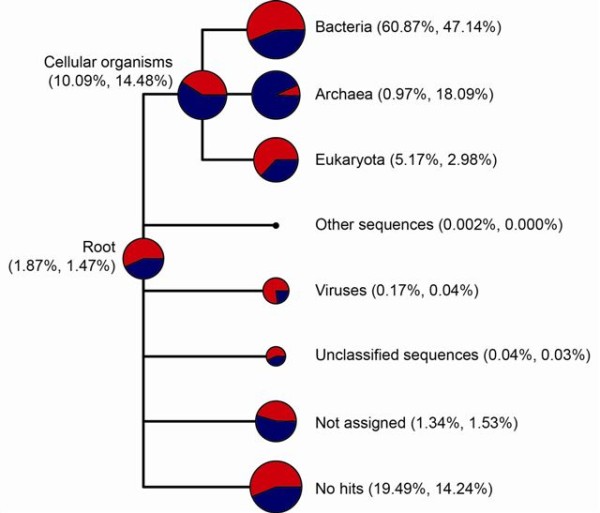
**Normalized MEGAN tree at the domain level**. Comparative tree view of the two metagenomes from the root to the domain level. The 0-4 cm metagenome is presented in red and the 10-15 cm metagenome in blue. The numbers in brackets give the percentage of total reads assigned to each node for the two metagenomes. The size of the individual nodes is scaled logarithmically to indicate number of reads assigned.

While most of the archaeal taxa in the 10-15 cm metagenome were accounted for, the number of taxa in the 0-4 cm was still increasing at 100% sampling. This difference is likely due to the low abundance of *Archaea *in the 0-4 cm metagenome (0.97% of reads) compared to the 10-15 cm metagenome (18.09% of reads) as shown in Figure 3.

### Taxonomic binning

There was a significant difference in the proportion of reads assigned to *Bacteria *and *Archaea *for the two metagenomes (Figure 3). In the 0-4 cm metagenome 60.87% of the reads were assigned to *Bacteria *and 0.97% to *Archaea*, while in the 10-15 cm metagenome 47.14% of the reads were assigned to *Bacteria *and as much as 18.09% to *Archaea*. This shift in the prokaryotic community structure suggests that *Archaea *thrive better and thereby also are likely to contribute more to the metabolism in the 10-15 cm sediment horizon.

Xipe analyses of the binned reads (confidence cut-off of 0.95, 0.98 and 0.99) at the phylum level (Table 1) and at the genus level (Additional file 2, Tables S2 and Additional file 3, Table S3) showed a significant difference between the two metagenomes as to the most abundant taxa [25]. The high abundance of *Archaea *in the 10-15 cm metagenome compared to the 0-4 cm metagenome was striking at the phylum level as well (Table 1). In the 10-15 cm metagenome, reads assigned to *Proteobacteria *and to "Archaeal environmental samples" were almost equally abundant, representing 15.45% and 13.03% of the reads respectively. In contrast, "Archaeal environmental samples" represented only 0.15% of the 0-4 cm metagenome, where reads assigned to *Proteobacteria *representing 31.07% were clearly most abundant (Table 1). *Euryarchaeota *was also significantly better represented in the 10-15 cm metagenome.

**Table 1:** Reads assigned to bacterial and archaeal taxa at the phylum-level in MEGAN

Domain	Phyla	0-4 cm metagenome		10-15 cm metagenome		Significant
		Reads assigned	Percent of reads	Reads assigned	Percent of reads	difference^1^
***Bacteria***	*Proteobacteria*	82318	31.07	30020	15.45	***
***Bacteria***	- *Gammaproteobacteria*^2^	27876	10.52	6442	3.31	***
***Bacteria***	- *Deltaproteobacteria*^2^	13777	5.20	12015	6.18	***
***Bacteria***	- *Alphaproteobacteria*^2^	8355	3.15	2416	1.24	***
***Bacteria***	- *Epsilonproteobacteria*^2^	5198	1.96	877	0.45	***
***Bacteria***	- *Betaproteobacteria*^2^	3045	1.15	1067	0.55	***
***Bacteria***	- *Zetaproteobacteria*^2^	282	0.11	77	0.04	***
***Bacteria***	*Bacteroidetes*	16782	6.34	6073	3.12	***
***Bacteria***	*Planctomycetes*	3657	1.38	2447	1.26	
***Bacteria***	*Firmicutes*	3620	1.37	4445	2.29	***
***Archaea***	*Euryarchaeota*	1353	0.51	6772	3.48	***
***Archaea***	Archaeal environmental samples	404	0.15	25317	13.03	***

The table presents number of reads assigned at the phylum level in MEGAN. For the phylum Proteobacteria, subsets of reads assigned proteobacterial classes are shown. All percentages are given as the percentage of total reads for each filtered metagenome. (Only phyla with at least 1% of the total unique reads in one or both samples are included.)

^1 ^*** indicates 99% confidence interval

**^2 ^**Reads assigned to *Proteobacteria *at the class level in MEGAN

Among the *Proteobacteria*, *Sulfurovum *was the most abundant genus in the 0-4 cm metagenome (Additional file 2, Table S2). This sulphur oxidizing genus, with its versatile energy metabolism, is known to thrive in sediments related to hydrothermal seepage where reductive and oxidative states in the mixing zone often fluctuate [26]. *Sulfurovum *was almost four times more abundant in the 0-4 cm metagenome compared to the 10-15 cm metagenome. This is consistent with oxidative zones being its preferred habitat [26].

### Taxa potentially involved in methane oxidation

**Figure 4:**
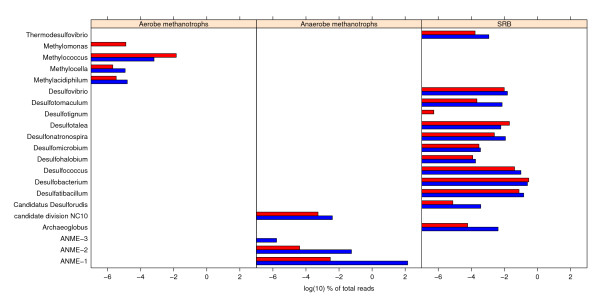
**Potential methanotrophic genera detected**. Shown is the proportion of reads assigned to methanotrophic genera at the genus level in MEGAN for each metagenome. In the left section known aerobic methanotrophic genera are presented. In the middle section known taxa involved in anaerobic methane oxidation are presented. In the right section known genera of sulphate reducing bacteria are presented. The archaeal sulphate reducing genus *Archaeoglobus *is also included in this section. The 0-4 cm metagenome is presented in red. The 10-15 cm metagenome is presented in blue. Numbers are given as log(10) percentage of total reads in each metagenome.

The methane oxidation measurements in the sediment cores indicated methanotrophic activity at both sediment depths. The metagenomes were searched for reads assigned to known methanotrophic genera that might be involved in methane oxidation. *Methylococcus *was the predominant aerobic methanotrophic genus in both metagenomes, but was significantly more abundant in the 0-4 cm metagenome where it accounted for 0.16% of the reads compared to the 10-14 cm metagenome where it accounted for 0.04% of the reads (Figure 4 and Additional file 2, Table S2). Although reads assigned to the aerobe methanotrophs *Methylomonas*, *Methylocella *and *Methylacidiphilum *were also detected, *Methylococcus *was approximately 10 and 2.5 times more abundant than these genera combined in the 0-4 cm and 10-15 cm metagenome respectively.

ANME groups were the predominant anaerobic methanotrophs in the sediments. Since taxonomic classification of reads in MEGAN was based on the NCBI taxonomy, the ANME clades were not recognized as independent taxa. The artificial taxon "Archaeal environmental samples" was however represented (Additional file 3, Table S3). Inspection of the reads assigned to this taxon revealed their assignment to ANME-1 and ANME-2 fosmids isolated from Eel River [11] or to "uncultured archaeon". Further inspection of the best hits for the reads assigned to "uncultured archaeon" (mean bit score 146.8) showed that most of these reads were associated to ANME as well, while a few reads were assigned to fosmids isolated from methane seeps offshore Japan [12,27-29] (Table 2).

**Table 2:** "Archaeal environmental samples"- reads assigned to ANME-sequences

Clade	0-4 cm metagenome		10-15 cm metagenome	
	Reads assigned	Percent of reads	Reads assigned	Percent of reads
ANME-1, Eel River [11,27]	27	0.01	3532	1.82
ANME-1, Black Sea [12]	177	0.07	12752	6.56
ANME-1b, Black Sea [28]	8	0.00	429	0.22
**Total ANME-1**	**212**	**0.08**	**16713**	**8.60**
ANME-2, Eel River [11]	20	0.01	534	0.27
ANME-2a [28]	11	0.00	14	0.01
ANME-2c [28]	2	0.00	12	0.01
**Total ANME-2**	**33**	**0.01**	**560**	**0.29**
ANME-3, Hydrate Ridge [28]	0	0.00	6	0.00
**Total ANME-3**	**0**	**0.00**	**6**	**0.00**

**Total ANME**	**245**	**0.09**	**17279**	**8.89**

The table presents reads assigned to Archaeal environmental samples" further classified as ANME. All percentages are given as the percentage of total reads for each filtered metagenome.

The ANME-1 clade was by far the anaerobic methanotroph with most assigned reads, although ANME-2 and ANME-3 also were present in the 10-15 cm metagenome (Figure 4). ANME-1 and ANME-2 were detected with low abundance in the 0-4 cm metagenome. The high abundance of ANME in the 10-15 cm metagenome indicates that AOM caused the high methane oxidation rates measured at this depth.

ANME are assumed to live in syntrophy with SRB. The most abundant genera of SRB in the metagenomes from the Tonya seep were *Desulfococcus*, *Desulfobacterium *and *Desulfatibacillum *(Figure 4). These genera were abundant in both metagenomes, and *Desulfococcus*, a common partner of ANME [7,9,10], especially so in the 10-15 cm metagenome (Additional file 2, Table S2).

Reads assigned to the bacterial NC10 group were present in both metagenomes (Figure 4). It has been proposed that *Candidatus Methylomirabilis oxyfera *of the NC10 group can oxidize methane anaerobically without an archaeal partner [30,31]. A pathway of "intra-aerobic" methane oxidation where an intracellular supply of oxygen is produced by metabolism of nitrite to oxygen and dinitrogen has been suggested. This intracellularly produced oxygen is then used for the oxidation of methane via *pmo*A [32]. Reads assigned to NC10 were significantly overrepresented (99% confidence interval) in the 10-15 cm metagenome compared to the 0-4 cm metagenome. Still, there was far less reads (approximately 1:100) assigned to NC10 than to ANME-1 in the 10-15 cm metagenome.

### Methane oxidation pathways

To gain insight into the metabolic pathways for methane oxidation at the Tonya Seep, we annotated the reads from each metagenome to KO and EC numbers and plotted them onto KEGG pathway maps. In this way, the methane monooxygenase gene (EC: 1.14.13.25) was identified in the 0-4 cm sample, supporting the idea of aerobic methane oxidation in this sediment horizon. This gene was not detected in the 10-15 cm metagenome.

**Figure 5:**
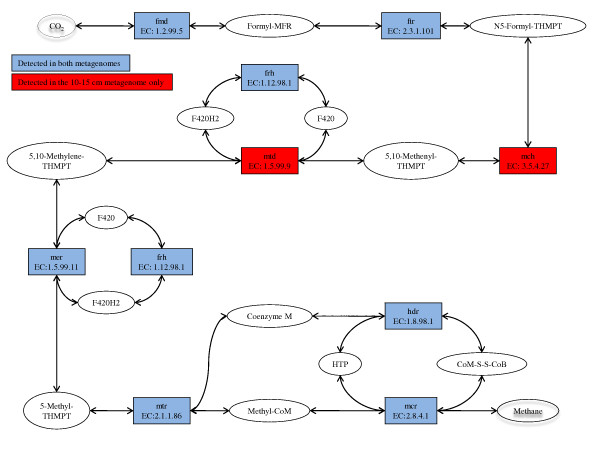
**Anaerobic oxidation of methane/methanogenesis pathway**. The figure is based on the KEGG-map for methane metabolism and includes the enzymes involved in methanogenesis and reverse methanogenesis. Colours are used to indicate from which metagenome the enzymes were identified by KAAS annotation.

All the genes needed for AOM/methanogenesis, including *mcrA *(EC: 2.8.4.1), were detected in the 10-15 cm metagenome (Figure 5). In the 0-4 cm metagenome, the genes for methylenetetrahydromethanopterin dehydrogenase (*mtd*, EC: 1.5.99.9) and methenyltetrahydromethanopterin cyclohydrolase (*mch*, EC: 3.5.4.27) were not detected. This is likely due to the low abundance of reads assigned to *Euryarchaeota *and "Archaeal environmental samples", and thereby low coverage of genes encoded by these taxa, in the 0-4 cm metagenome. In total, 1757 reads were assigned to these taxa in the 0-4 cm metagenome. With an average sequence length of 413 bases this gives a total of 0.7 M bases, while the average ANME-1 genome size is estimated to be 3.3-3.6 Mbp (Table 1) [12].

Anaerobic oxidation of methane is usually associated with dissimilatory sulphate reduction, where adenylyl-sulphate reductase (EC: 1.8.99.2) first reduces sulphate to sulphite before dissimilatory sulphite reductase (EC: 1.8.99.3) reduces sulphite to sulphide [13]. These genes were detected in both metagenomes.

### Marker genes

**Figure 6:**
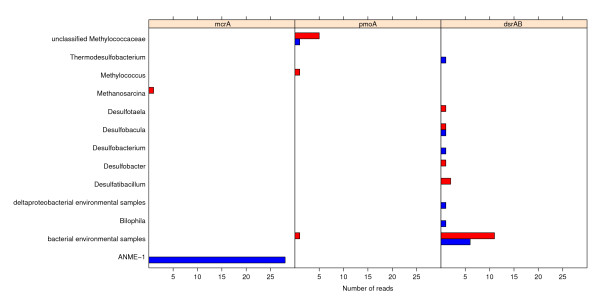
**Taxonomic distribution of marker genes for methane oxidation**. Shown is the number of reads matching marker genes associated with oxidation of methane and the taxonomic distribution of these reads in each metagenome. Reads matching the marker genes for anaerobic oxidation of methane (*mcr*A), aerobic oxidation of methane (*pmo*A) and sulphate reduction (*dsr*AB) are presented in the left, middle and right section respectively. The 0-4 cm metagenome is presented in red and the 10-15 cm metagenome in blue.

To obtain a more precise picture of taxa actually capable of methane oxidation in our sediment, the metagenomes were compared with libraries of marker genes for methane oxidation. Estimated probabilities for identifying the specific marker genes were used to calculate expected hits to marker genes in a scenario where all organisms in the communities contained the gene in question (Additional file 1, Table S1). Based on these expected numbers, and the number of marker genes actually detected, we estimated the fraction of the community containing the gene. Eight reads in total matched *pmo*A, the marker gene for aerobic methane oxidation (Figure 6). In MEGAN, one of these was assigned to the genus *Methylococcus *of the family *Methylococcaceae *while six reads were assigned to unclassified *Methylococcaceae*. This point towards *Methylococcaceae *as the most important family of aerobic methane oxidizers at the Tonya seep sediments, as was also indicated by taxonomic abundance. Seven out of eight reads assigned to *pmo*A were from the 0-4 cm sample, supporting that aerobic methane oxidation is conducted in the shallower layer of the sediment. The estimated fraction of the community coding for *pmo*A, based on marker gene detection, was calculated to 12.9% and 1.5% in the 0-4 cm and 10-15 cm respectively (Additional file 1, Table S1).

The marker gene for AOM, *mcr*A, is also a key gene in methanogenesis, where it catalyzes the last step. The 0-4 cm sample contained only one *mcr*A read, assigned to the methanogenic genus *Methanosarcina *(Figure 6). In the 10-15 cm sample 28 reads matching *mcr*A were found, all assigned to ANME-1. Based on EGS and expected number of reads matching *mcr*A, the estimated fraction of the community in the 10-15 cm sample made up of ANME-1 was 77.4% (Additional file 1, Table S1).

In order to detect possible SRB partners of ANME, we compared the two metagenomes to a *dsr*AB library. Of 60 hits, 33 were assigned to the reversed form of *dsr*AB found in sulphur compound-oxidizing bacteria. Sixteen and eleven *dsr*AB reads from the possible SRB partners of ANME were detected in the 0-4 cm and 10-15 cm metagenomes respectively, estimations based on the probability of detecting this gene thereby indicate that 43.2% and 24.6% of the 0-4 cm and 10-15 cm community were made up by SRB respectively (Additional file 1, Table S1).

Most SRB *dsr*AB reads were assigned to "bacterial environmental samples" and the deltaproteobacterial genera *Desulfotaela*, *Desulfobacula*, *Desulfobacterium*, *Desulfobacter*, *Desulfatibacillum *and *Bilophila *(Figure 6). The reads assigned to "bacterial environmental samples" matched clones from a diverse range of sediments [33-41] and one clone from an acidic fan soil sample [42]. Eight of the reads assigned to "bacterial environmental samples" (six from the 0-4 cm metagenome and two from the 10-15 cm metagenome) were most similar to *dsr*AB sequences from *Deltaproteobacteria *[33-39] (results not shown). None of the *dsr*AB reads were assigned to *Desulfosarcina *or *Desulfococcus*, the previously described syntrophic partners of ANME-1 [7,9,10].

## Discussion

### Methane oxidation rate

Methane oxidation rates in our sediment cores were 156 ± 64 nmol cm^-3 ^day^-1^. This is much higher than the methane oxidation rates at the nearby Brian seep (6-87 nmol cm^-3 ^day^-1^) [24] and within the range of AOM at seeps with surface hydrates, mud volcanoes and gas chimneys ([13] and refs therein). It has been suggested that the relatively low methane oxidation rate at the Brian seep could be caused by the permeable, sandy sediments leading to low amounts of dissolved methane in the pore water [24]. Conversely, the higher methane oxidation rate at the Tonya seep could be due to the less permeable, relatively oily tar containing sediments at this seep.

### Taxonomic richness and coverage

Taxonomic classification was based on a blastX query against the NCBI non-redundant Protein Database (ncbiP-nr). It has previously been shown that the prokaryotic representation in public sequence databases, such as the ncbiP-nr, is heavily biased towards taxa that are easily cultivable or of anthropogenic interest [43,44]. Many of the taxa represented are further only partially sequenced [44]. These issues may lead to false assignment of reads, especially if only the top hit is considered. By employing the LCA algorithm of MEGAN, most of these wrong assignments are avoided at the cost of more reads being assigned to taxa of low specificity or not being assigned at all [45,46]. Short reads may also be a source of ambiguous taxonomic classification, especially if they are from a highly conserved region of the genome or from a region susceptible to horizontal gene transfer [44,45,47]. We therefore calculated the average read length for reads assigned to different taxonomic levels in MEGAN to see if it decreased with decreasing taxonomic specificity (Additional file 4, Table S4). This was not the case as average lengths of reads assigned to all taxonomic levels in MEGAN (including "not assigned") were in the same range (approximately 450 bases). Read with no hits against the ncbiP-nr were however considerably shorter (average read lengths of 263 ± 181 and 232 ± 175 bases in 0-4 cm and 10-15 cm metagenome respectively).

Rarefaction analyses indicated that the most abundant taxa of the Tonya Seep sediments were accounted for in our metagenomes. The taxonomic richness of prokaryotes, in combination with high EGS, does however lead to low coverage of most genomes represented in the metagenomes. Absence of a single marker gene assigned to a specific taxon might therefore be due to chance. Still, we detected more marker genes than expected based on the taxonomic binning of reads. This could be due to an overestimation of the EGS. It has previously been discussed that a bit score threshold of 60 (as used in this work according to the method developed by Raes *et al*. [48]) might discriminate against short reads, and that lowering of the threshold would result in decreased EGS [49]. A decreased EGS would in turn result in a reduction of the estimated fraction of the community carrying the marker genes *mcr*A, *pmo*A and *dsr*AB. Differences in copy number for organisms carrying the gene might also affect the expected number of hits.

### Aerobic methane oxidation

Due to limited oxygen penetration, active aerobic methane oxidation is probably limited to a thin surface layer. The maximum oxygen penetration at the nearby Brian seep sediments was measured to a depth of 1.4 cm [24]. Due to high tar content, oxygen penetration in the sediments of the Tonya seep is expected to be more restricted than at the Brian seep.

Methane monooxygenase (EC: 1.14.13.25) was only detected in the 0-4 cm metagenome after plotting of KO and EC numbers onto KEGG pathway maps. Overrepresentation of aerobic methanotrophic genera and *pmo*A (based on library comparison) in the 0-4 cm metagenome compared to the 10-15 cm metagenome further support aerobic oxidation of methane in the 0-4 cm sediment sample (see Figures 4 and 6).

Both taxonomic binning of reads and marker gene classification point to type I methanotrophs of *Methylococcaceae *as the most important aerobic methane oxidizers in our samples. While *Methylococcus *was the aerobic methanotrophic genus with most reads assigned (see Figure 4), most of the detected *pmo*A reads were assigned to unclassified *Methylococcaceae *(see Figure 6). This indicates that uncultured type I methanotrophs might play an important role in aerobic methane oxidation at the Tonya Seep. Also in microbial mats and sediments of the nearby Shane and Brian seeps aerobic type I methanotrophs have been identified, while no type II methanotrophs were detected at either of these sites [21,22]. This is consistent with type I methanotrophs dominating over type II methanotrophs in most marine settings ([50]and refs therein).

### Anaerobic methane oxidation

Genes for AOM were detected in both metagenomes (see Figure 5). The taxonomic binning of reads points to AMNE-1 as the predominant anaerobic oxidizer of methane in the Tonya seep sediment, especially in the 10-15 cm sediment sample. It is however, important to notice that ANME-1, due to the genome sequencing efforts [12], is the most sequenced ANME-clade, and therefore overrepresented in the database. This could skew our relative abundance results. However, the presence and dominance of ANME-1 was further supported by the *mcrA *reads in our metagenomes (see Figure 6). This gene is identified in all ANME-clades, still all reads matching *mcr*A in the 10-15 cm metagenome were assigned to ANME-1. Taken together, these results provide strong evidence of ANME-1 being the most important clade for anaerobic methane oxidation in the Tonya seep sediments. In contrast, only ANME-2 was detected at the nearby Brian Seep [24]. ANME-1 and ANME-2 are known to co-occur in sediments, usually with one type more abundant than the other [7,51,52]. The environmental conditions that might regulate the relative abundance of the different ANME clades in marine sediments are still not known [7,51]. Differences in permeability of the sediments at the Tonya and Brian seeps could be one factor selecting for different ANME clades at the two sites.

### Sulphate reducing bacteria

Anaerobic oxidation of methane is assumed to be coupled to dissimilatory reduction of sulphate. Both metagenomes had reads assigned to SRB genera, predominantly *Desulfococcus*, *Desulfobacterium *and *Desulfatibacillum *(see Figure 4). The ratio of total reads assigned to ANME related to reads assigned to each of these SRB genera in the 10-15 cm metagenome were ANME: *Desulfobacterium*; 16: 1, ANME *Desulfatibacillum*; 20:1 and ANME: *Desulfococcus*; 24: 1. The total ratio ANME: SRB (including "Bacteria environmental samples") was 4: 1.

Reads assigned to *dsr*AB were detected in both metagenomes and classified to a diverse set of taxa (see Figure 6). Although the fraction of the community containing *mcr*A and *dsr*AB, calculated based on sampling probability of the specific marker genes, is likely to be overestimated it gives a similar ratio of 3: 1 of mcrA-containing organisms: dsrAB containing organisms as the taxonomic binning of reads. None of our *dsr*AB reads were assigned to the known ANME partner *Desulfococcus*, although this genus was one of the most abundant SRB genera in our metagenomes (see Figure 4). This does not imply absence of *dsr*AB among *Desulfococcus *in our samples; the gene was more likely missed by chance due to low coverage (see Additional file 2, Table S2).

ANME might also form syntrophic relationships to other bacteria than those most commonly recognized. ANME-2 has previously been detected to form physical associations to both *Desulfobulbus *and a member of the *Betaproteobacteria*, as well as their regular partners from the *Desulfococcus/Desulfosarcina *branch [53]. The main bulk of *dsr*AB-reads in the 10-15 cm metagenome were assigned to "bacterial environmental samples" and the ANME partners might be found among these organisms. The "bacterial environmental samples" is however a diverse group and was also abundant in the 0-4 cm metagenome, where ANME were less abundant.

Our results do not indicate only one predominant ANME partner, but rather that several syntrophic partners may be involved. Diverse *dsr*AB signatures with only weak coupling to AOM have previously been detected in ANME-1 dominated sediments in the Gulf of Mexico [39]. This suggests that these seep environments have a high diversity of taxa involved in sulphate reduction.

## Conclusions

By using 454 sequenced metagenomes we achieved an insight into the taxonomic richness of the seep sediments. Most of the taxa were present in both metagenomes and differences in community structure and corresponding metabolic potential between the two samples were due to abundance, indicating sliding boundaries between the different communities. Our approach provided strong evidence for the taxa responsible for methane oxidation. The Tonya Seep harboured several taxa potentially capable of methane oxidation under both aerobic and anaerobic conditions. This suggests that the sediment is a robust methane filter, where taxa presently dominating this important process could be replaced by less abundant taxa should the environmental conditions change.

## Methods

### Sampling site

Tonya Seep (34°24.043'N; 119°52.841'W) is located in the Coal Oil Point seep field offshore Santa Barbara, California, USA. Tonya Seep is primarily a single 2 m diameter pit with many vents inside that rapidly coalesce into a single plume. There was a high content of hydrocarbons and tar in the sediments. Four sediment cores, two for methane oxidation studies and two for metagenomic analysis, were collected at 25 m depth on July 16^th ^2008 by UC Santa Barbara Marine Operation divers. The polycarbonate liners used (30 cm length and 3.5 cm diameter) were treated with 70% ethanol and dried before sampling. The parallel cores (core I, II, III and IV) were sealed at the seafloor and kept on ice during transportation back to shore.

### Gas Sample Collection

Two seep gas samples (Gas samples I and II) were collected in the surface waters above the seep. The samples were collected on two occasions from small vessels via an inverted funnel method in which seep gas bubbles were captured into 120 mL glass serum vials after rising through the water column. Bottles were capped underwater after filling to avoid contamination with atmospheric gases. Seep gases were analyzed by gas chromatography as previously described [54]. Error associated with the concentration measurements was ±4%.

### Methane oxidation rates

Cores III and IV designated for methane oxidation rate (MOR) measurements were injected with radiotracer ^14^C-CH_4 _(1 kBq ^14^CH_4 _dissolved in water, 20 μL injection volume) at 2 cm intervals and incubated at near *in-situ *temperature. After 18 hours the core was sub-sectioned and placed into vials with 1 M NaOH and quickly sealed, ending the incubation and trapping the CO_2_. A small sample of headspace (0.2 mL) was removed to determine CH_4 _concentration (which is not affected by the ^14^CH_4 _spike) by GC-FID (Shimadzu GC-4A, 6 ft length 80/100 mesh Molsieve 13X packed column run isothermally at 140°C with N_2 _carrier flow at 15 mL min^-1^). The remaining ^14^CH_4 _in the headspace of the vial was purged via a slow flow of air through a combustion tube filled with Cu(II)-oxide and maintained at 850°C. The resulting ^14^CO_2 _was trapped using a mixture of phenethylamine and 2-methoxyethanol. The remaining ^14^CO_2_, which was assumed to be microbially produced, was measured by first transferring the sediment into a 100 mL Erlenmeyer flask fitted with a small (7 mL) phenethylamine/NaOH-filled scintillation vial suspended beneath its rubber stopper. Six ml of hydrochloric acid (6 M) was injected through the rubber stopper to degas the CO_2 _from the sediment/NaOH slurry, and the flask was placed in a shaker for ~8 hrs to transfer the CO_2 _to the suspended scintillation vial. Radioactivity was quantified by scintillation counting (Beckman LSC 6500).

The *ex-situ *CH_4 _oxidation rates (MOR) were calculated by the following equation:

(1) MOR =C14O2×CH4/(C14H4×v×t)

where ^14^CO_2 _is the activity of the microbially-produced CO_2_, CH_4 _is the amount of CH_4 _in the sample, ^14^CH_4 _is the activity of the injected CH_4_, v is the volume of the sediment and t is the incubation time.

### DNA extraction

For metagenomic analysis, cores I and II were pushed out from the liners and the 0-4 cm bsf and the 10-15 cm bsf horizons were removed for DNA extraction. Multiple parallel 0.5 g subsamples of the cores at each horizon were used for DNA extraction. Total genomic DNA was extracted with a FastDNA^®^SPIN for Soil Kit (MP Biomedicals) and cleaned using Wizard DNA Clean-Up (Promega) according to the manufacturer's instructions. The DNA quality was assessed by agarose gel electrophoresis and by optical density using a NanoDrop instrument (NanoDrop Products, Thermo Scientific). To get enough high quality DNA for the subsequent 454 sequencing DNA, subsamples from the same horizon were pooled. Of the total DNA isolated from the 0-4 cm horizon, 35% originated from core I and 65% from core II. For the 10-15 cm horizon, 38% was isolated from core I and 62% from core II.

### 454 sequencing

For creation of the metagenomic libraries, 9.8 μg DNA of the 0-4 cm sample and 6.8 μg of the 10-15 cm sample were used. Sample preparation and sequencing of the extracted DNA were performed at the Norwegian High-Throughput Sequencing Centre (NSC) at CEES [55], University of Oslo according to standard GS FLX Titanium protocols, except that after the initial dsDNA immobilization, ssDNA was brought into solution by adding 50 μl 1 × TE to the beads, followed by 2 min at 90°C and rapid cooling on ice.

The samples were tagged (fusion primers with tag sequences were used to mark sample origin), mixed and sequenced on a 70 × 75 format PicoTiterPlate™ on a GS FLX titanium instrument.

The metagenomic reads have been submitted to the Genbank Sequence Read archive [GeneBank: SRP005641].

The average of the mean quality score per sequence was 33.1 (standard deviation: 3.6) and 32.9 (standard deviation: 3.5) for the 0-4 cm metagenome and 10-15 cm metagenome respectively.

### Replicate removal

Replicate reads were removed from the two metagenomes using the 454 Replicate filter [56,57]. Standard settings of a sequence identity cut off of 0.9, a length difference requirement of 0 and a number of beginning base pairs to check of 3, were used. After removal of replicates, the 0-4 cm metagenome contained 525 reads with more than 2 ambiguous bases and 1222 reads with long homopolymers (> 10 nt), making a total of 1733 (0.65%) low quality reads. The 10-15 cm metagenome contained 395 reads with more than 2 ambiguous bases and 143 reads with long homopolymers (> 10 nt), making a total of 535 (0.28%) low quality reads.

### Taxonomic classification

The reads were taxonomically classified by BlastX query against the NCBI non-redundant Protein Database (ncbiP-nr) [58]. The computation was performed at the freely available Bioportal computer service [59]. Maximum expectation-value was set to 10.0 and maximum 25 alignments were reported per hit. The BlastX output files were analysed according to NCBI taxonomy in the program MEGAN, version 3.9 [44] with default LCA-parameters (Min Score: 35, Top Percent: 10.0 and Min Support: 5). We used the option "enable all taxa" in MEGAN in order to account for reads with hits to the artificial taxa archaeal and bacterial "environmental samples".

### Rarefaction analysis

The species richness was estimated by rarefaction analysis performed in MEGAN [44]. The MEGAN program uses an LCA-algorithm to bin reads to taxa based on their blast-hits. This results in a rooted tree where each node represents a taxon. The leaves in this tree are then used as OTUs in the rarefaction analysis. The program randomly chooses 10%, 20% ... 100% of the total number of reads as subsets. For each of these random subsets the number of leaves (hit with at least 5 reads (Min Support) is determined. This sub sampling is repeated 20 times and then the average value is used for each percentage. We did the analysis at the most resolved level of the NCBI taxonomy to capture as much of the richness as possible. At this level, the leaves are mostly strains and species but also some sequences like fosmids and plasmids are included. In cases were no reads are assigned to species the most detailed taxonomic level with 5 reads or more assigned are used.

The analysis was performed for total taxa in the metagenomes (including *Bacteria*, *Archaea*, *Eukaryota*, Viruses and Environmental sequences), and separately for archaeal and bacterial taxa.

### Comparison of metagenomes

The metagenomes were compared at the phylum, class and genus level in MEGAN using absolute read counts [44]. Tabulated text files for each level were extracted from MEGAN and analyzed in the following manner: The metagenomes were normalized to the size of the smallest metagenome. Taxa without matches in one metagenome, or with less than 20 reads in both metagenomes, were removed from the comparison since they (due to their low abundance) could have been identified by chance and thereby represent uninformative data. The resulting normalized comparison was analyzed for overrepresented taxa using XIPE-totec with 20.000 samplings and with a confidence cut-off of 0.95, 0.98 and 0.99 [25].

### Metabolic potential

Reads were annotated to KEGG Orthologe (KO)-identifiers using KEGG Automatic Annotation Server (KAAS) [60,61]. Parameters used were: single-directional best hit, default bit score (60) and 40 manually selected reference genomes (Additional file 5, Table S5). Reference genomes were chosen from the most abundant species present in the metagenomes based on annotation in MEGAN.

The KO-identifiers were, if possible, replaced by corresponding Enzyme Commission (EC)-numbers using the Kyoto Encyclopedia of Genes and Genomes (KEGG) Orthology database [62-65].

Lists of unique EC and KO numbers (when no EC-number was obtained) were created for each metagenome. These lists were then used to plot metabolic pathways for the two metagenomes onto metabolic pathway maps using KEGG Mapper: Colour Objects in KEGG Pathways [62-65].

### Signature genes for methane oxidation

The reads were compared to protein sequence libraries for methyl-coenzyme M reductase (*mcr*A), particulate methane monooxygenase (*pmo*A) and dissimilatory sulphite reductase (*dsr*AB) on the freely available Bioportal computer service [59]. The reference library for each enzyme was downloaded from Fungene (Functional gene pipeline & repository) version v6.1 [66]. We limited the libraries by selecting only the sequences with a score (bits saved) of 100 or more from the HMMER Hidden Markov Model search against NCBIs non-redundant protein database. We used blastX against the protein sequences of each enzyme library with a maximum expectation value of 1.0E-20 [58]. Maximum one alignment was reported.

BlastX output files were further analyzed using NCBI-taxonomy in MEGAN, version 3.9 [44]. The LCA-parameters were set to: Min Score: 35, Top Percent: 10.0 and Min Support: 1. All taxa were enabled.

### Estimates of effective genome sizes (EGS) and sampling probabilities of individual genes

EGS was calculated according to the method developed by Raes *et al *[48] using the parameters a = 18.26, b = 3650 and c = 0.733. Blast against a subset of the STRING database (v9.0), containing the COGs concerned, were conducted at the freely available Bioportal computer service [59,67].

Sampling probability of the individual marker genes and expected number of sequences detected was calculated according to Beszteri et al [68]. We calculated with an average copy number of two for *pmo*A [69] and one for *mcr*A and *dsr*AB [70-72]. Average marker gene length was based on the reads present in the respective marker gene databases.

## Authors' contributions

OEH participated in the design of the study carried out the taxonomic, marker gene and pathway analyses and drafted the manuscript. THAH participated in the design of the study and performed the statistical analysis. TK and KSJ participated in the design of the study. AGR conceived the study, participated in its design and isolated DNA from the sediment samples acquired during her stay in David Valentines group at the University of California Santa Barbara. All authors helped revise the manuscript. All authors read and approved the final manuscript.

## Supplementary Material

Additional file 1 **Table S1**. Calculations based on estimated Effective Genome Sizes. (References are listed in the reference list of the main manuscript). Click here for file

Additional file 2 **Table S2**. Reads assigned to bacterial taxa at the genus level in MEGAN (more than 0.1% of total reads assigned in at least one of the samples). All percentages are given as the percentage of total reads for each filtered metagenome. Click here for file

Additional file 3 **Table S3**. Reads assigned to archaeal taxa at the genus level in MEGAN (more than 0.1% of total reads assigned in at least one of the samples). All percentages are given as the percentage of total reads for each filtered metagenome. Click here for file

Additional file 4 **Table S4**. Reads length distribution for reads assigned at different taxonomic levels in MEGAN. Click here for file

Additional file 5 **Table S5**. Genomes used for KAAS annotation. Click here for file
