# Supplementary material for: A metagenomic study of methanotrophic microorganisms in Coal Oil Point seep sediments
Source: BMC Microbiol. 2011 Oct 4;11:221. doi: 10.1186/1471-2180-11-221 (PMC3197505; doi:10.1186/1471-2180-11-221)
Supplement: Additional file 1 — Table S1. Calculations based on estimated Effective Genome Sizes. (References are listed in the reference list of the main manuscript). [file 1471-2180-11-221-S1.DOC]

**Table** **S1:** Calculations based on estimated Effective Genome Sizes

(References are listed in the reference list of the main manuscript)

| **Parametres** | **0-4 cm** | **10-15 cm** |
| --- | --- | --- |
| **EGS: (a+b∙L^-c^)/x**  **Where a, b and c are constants, L is average read length and x is marker gene density** | 4.8 Mbp | 4.0 Mbp |
| **Length marker gene (l_mcrA_)** | 542 | 542 |
| **Length marker gene (l_pmoA_)** | 490 | 490 |
| **Length marker gene (l_dsrAB_)** | 670 | 670 |
| **Average copy number (C_mcrA_) in the community** | 1 | 1 |
| **Average copy number (C_pmoA_) in the community** | 2 | 2 |
| **Average copy number (C_dsrAB_) in the community** | 1 | 1 |
| **Probability of detecting mcrA:**  **p_mcrA_=(l_mcrA_/EGS) ∙ C_mcrA_** | 0.000113 | 0.000137 |
| **Probability of detecting pmoA:**  **p_pmoA_=( l_pmoA_ /EGS) ∙ C_pmoA_** | 0.000204 | 0.000247 |
| **Probability of detecting dsrAB:**  **p_dsrAB_=(l_dsrAB_ /EGS) ∙ C_dsrAB_** | 0.000139 | 0.000169 |
| **Expected hits to mcrA (assuming it is present in all organisms): n_mcrA_= p_mcrA_ ∙ Number of reads in metagenome** | 30 | 36 |
| **Expected hits to pmoA (assuming it is present in all organisms): n_pmoA_= p_pmoA_ ∙ Number of reads in metagenome** | 54 | 66 |
| **Expected hits to dsrAB (assuming it is present in all organisms): n_dsrAB_= p_dsrAB_ ∙ Number of reads in metagenome** | 37 | 45 |
| **Number of mcrA detected (N_mcrA_)** | 1 | 28 |
| **Number of pmoA detected (N_pmoA_)** | 7 | 1 |
| **Number of dsrAB detected (N_dsrAB_)** | 16 | 11 |
| **Estimated fraction of community containing mcrA:**  **(N_mcrA_ / n_mcrA_) ∙ 100** | 3.34 % | 77.37 % |
| **Estimated fraction of community containing pmoA:**  **(N_pmoA_ / n_pmoA_) ∙ 100** | 12.91 % | 1.53% |
| **Estimated fraction of community containing dsrAB:**  **(N_dsrAB_ / n_dsrAB_) ∙ 100** | 43.22 % | 24.59 % |
