# Supplementary material for: A metagenomic study of methanotrophic microorganisms in Coal Oil Point seep sediments
Source: BMC Microbiol. 2011 Oct 4;11:221. doi: 10.1186/1471-2180-11-221 (PMC3197505; doi:10.1186/1471-2180-11-221)
Supplement: Additional file 2 — Table S2. Reads assigned to bacterial taxa at the genus level in MEGAN (more than 0.1% of total reads assigned in at least one of the samples). All percentages are given as the percentage of total reads for each filtered metagenome. [file 1471-2180-11-221-S2.DOC]

**Table S2:**

Reads assigned to bacterial taxa at the genus level in MEGAN (more than 0.1% of total reads assigned in at least one of the samples). All percentages are given as the percentage of total reads for each filtered metagenome.

| **Phyla/ class** | **Genera** | **0-4 cm** | | **10-15 cm** | | **Significant** |
| --- | --- | --- | --- | --- | --- | --- |
|  |  | **Reads** | **Percent** | **Reads** | **Percent** | **difference^1^** |
| ***Acidobacteria*** | *Candidatus Solibacter* | 304 | 0.11 | 226 | 0.12 |  |
| ***Actinobacteria*** | *Streptomyces* | 281 | 0.11 | 172 | 0.09 |  |
| ***Bacteroidetes*** | unclassified Flavobacteriales (miscellaneous) | 1511 | 0.57 | 343 | 0.18 | *** |
| ***Bacteroidetes*** | *Bacteroides* | 656 | 0.25 | 385 | 0.20 | *** |
| ***Bacteroidetes*** | *Polaribacter* | 472 | 0.18 | 84 | 0.04 | *** |
| ***Bacteroidetes*** | *Microscilla* | 466 | 0.18 | 229 | 0.12 | *** |
| ***Bacteroidetes*** | *Rhodothermus* | 192 | 0.07 | 207 | 0.11 |  |
| ***Betaproteobacteria*** | *Burkholderia* | 296 | 0.11 | 153 | 0.08 | ** |
| ***Chlorobi*** | *Chloroherpeton* | 174 | 0.07 | 224 | 0.12 |  |
| ***Chloroflexi*** | *Roseiflexus* | 192 | 0.07 | 424 | 0.22 | *** |
| ***Chloroflexi*** | *Chloroflexus* | 100 | 0.04 | 221 | 0.11 | *** |
| ***Chloroflexi*** | *Dehalococcoides* | 63 | 0.02 | 295 | 0.15 | *** |
| ***Cyanobacteria*** | *Cyanothece* | 235 | 0.09 | 205 | 0.11 |  |
| ***Deltaproteobacteria*** | *Desulfobacterium* | 1570 | 0.59 | 1062 | 0.55 | ** |
| ***Deltaproteobacteria*** | *Geobacter* | 1101 | 0.42 | 900 | 0.46 |  |
| ***Deltaproteobacteria*** | *Desulfatibacillum* | 869 | 0.33 | 847 | 0.44 |  |
| ***Deltaproteobacteria*** | *Desulfococcus* | 664 | 0.25 | 715 | 0.37 | ** |
| ***Deltaproteobacteria*** | *Desulfotalea* | 488 | 0.18 | 212 | 0.11 | *** |
| ***Deltaproteobacteria*** | *Syntrophobacter* | 407 | 0.15 | 497 | 0.26 | *** |
| ***Deltaproteobacteria*** | *Desulfovibrio* | 356 | 0.13 | 317 | 0.16 |  |
| ***Deltaproteobacteria*** | *Pelobacter* | 342 | 0.13 | 218 | 0.11 |  |
| ***Deltaproteobacteria*** | *Desulfuromonas* | 334 | 0.13 | 111 | 0.06 | *** |
| ***Deltaproteobacteria*** | *Haliangium* | 320 | 0.12 | 146 | 0.08 | *** |
| ***Deltaproteobacteria*** | *Syntrophus* | 232 | 0.09 | 536 | 0.28 | *** |
| ***Deltaproteobacteria*** | *Desulfonatronospira* | 196 | 0.07 | 281 | 0.14 | *** |
| ***Epsilonproteobacteria*** | *Sulfurovum* | 2289 | 0.86 | 456 | 0.23 | *** |
| ***Epsilonproteobacteria*** | unclassified Campylobacterales | 574 | 0.22 | 78 | 0.04 | *** |
| ***Gammaproteobacteria*** | unclassified Gammaproteobacteria (miscellaneous) | 1768 | 0.67 | 473 | 0.24 | *** |
| ***Gammaproteobacteria*** | *Thioalkalivibrio* | 998 | 0.38 | 162 | 0.08 | *** |
| ***Gammaproteobacteria*** | *Nitrosococcus* | 786 | 0.30 | 256 | 0.13 | *** |
| ***Gammaproteobacteria*** | *Beggiatoa* | 563 | 0.21 | 316 | 0.16 | *** |
| ***Gammaproteobacteria*** | *Endoriftia* | 509 | 0.19 | 114 | 0.06 | *** |
| ***Gammaproteobacteria*** | *Shewanella* | 475 | 0.18 | 142 | 0.07 | *** |
| ***Gammaproteobacteria*** | *Methylococcus* | 419 | 0.16 | 80 | 0.04 | *** |
| ***Gammaproteobacteria*** | *Methylophaga* | 414 | 0.16 | 56 | 0.03 | *** |
| ***Gammaproteobacteria*** | *Vibrio* | 309 | 0.12 | 136 | 0.07 | *** |
| ***Gammaproteobacteria*** | *Hahella* | 267 | 0.10 | 66 | 0.03 | *** |
| ***Gammaproteobacteria*** | *Pseudomonas* | 265 | 0.10 | 51 | 0.03 | *** |
| ***Gammaproteobacteria*** | *Allochromatium* | 262 | 0.10 | 85 | 0.04 | *** |
| ***Zetaproteobacteria*** | *Mariprofundus* | 282 | 0.11 | 77 | 0.04 | *** |
| ***Firmicutes*** | *Clostridium* | 599 | 0.23 | 575 | 0.30 |  |
| ***Firmicutes*** | *Bacillus* | 281 | 0.11 | 274 | 0.14 |  |
| ***Firmicutes*** | *Desulfotomaculum* | 68 | 0.03 | 230 | 0.12 | *** |
| ***Lentisphaerae*** | *Lentisphaera* | 370 | 0.14 | 152 | 0.08 | *** |
| ***Planctomycetes*** | *Planctomyces* | 699 | 0.26 | 388 | 0.20 | *** |
| ***Planctomycetes*** | *Rhodopirellula* | 633 | 0.24 | 315 | 0.16 | *** |
| ***Planctomycetes*** | *Pirellula* | 317 | 0.12 | 157 | 0.08 | *** |
| ***Planctomycetes*** | *Blastopirellula* | 303 | 0.11 | 187 | 0.10 |  |
| ***Planctomycetes*** | *Candidatus Kuenenia* | 300 | 0.11 | 537 | 0.28 | *** |
| ***Verrucomicrobia*** | Verrucomicrobia subdivision 3 | 256 | 0.10 | 191 | 0.10 |  |
| ***unclassified Bacteria*** | *Candidatus Cloacamonas* | 210 | 0.08 | 539 | 0.28 | *** |
| **Bacteria environmental samples** | | 280 | 0.11 | 203 | 0.10 |  |

^1^ The confidence level of significant differences is indicated by asterisks. ** indicates a 98% confidence interval, while *** indicates a 99% confidence interval.
