# Supplementary material for: A metagenomic study of methanotrophic microorganisms in Coal Oil Point seep sediments
Source: BMC Microbiol. 2011 Oct 4;11:221. doi: 10.1186/1471-2180-11-221 (PMC3197505; doi:10.1186/1471-2180-11-221)
Supplement: Additional file 3 — Table S3. Reads assigned to archaeal taxa at the genus level in MEGAN (more than 0.1% of total reads assigned in at least one of the samples). All percentages are given as the percentage of total reads for each filtered metagenome. [file 1471-2180-11-221-S3.DOC]

**Table S3:** Reads assigned to archaeal taxa at the genus level in MEGAN (more than 0.1% of total reads assigned in at least one of the samples). All percentages are given as the percentage of total reads for each filtered metagenome.

| **Phyla** | **Genera** | **0-4 cm** | | **10-15 cm** | | **Significant differnce^1^** |
| --- | --- | --- | --- | --- | --- | --- |
|  |  | **Reads** | **Percent** | **Reads** | **Percent** |  |
| ***Euryarchaeota*** | *Methanosarcina* | 164 | 0.06 | 923 | 0.47 | *** |
| ***Euryarchaeota*** | *Thermococcus* | 57 | 0.02 | 282 | 0.15 | *** |
| ***Euryarchaeota*** | *Methanococcoides* | 49 | 0.02 | 189 | 0.10 | *** |
| ***Euryarchaeota*** | *Aciduliprofundum* | 40 | 0.02 | 226 | 0.12 | *** |
| ***Euryarchaeota*** | *Ferroglobus* | 39 | 0.01 | 242 | 0.12 | *** |
| **Archaea environmental samples** | | 404 | 0.15 | 25317 | 13.03 | *** |

^1^ *** indicates a 99% confidence interval.
