# Supplementary material for: A metagenomic study of methanotrophic microorganisms in Coal Oil Point seep sediments
Source: BMC Microbiol. 2011 Oct 4;11:221. doi: 10.1186/1471-2180-11-221 (PMC3197505; doi:10.1186/1471-2180-11-221)
Supplement: Additional file 4 — Table S4. Reads length distribution for reads assigned at different taxonomic levels in MEGAN. [file 1471-2180-11-221-S4.DOC]

**Table S4:** Reads length distribution for reads assigned at different taxonomic levels in MEGAN

|  |  |  | **Read length distribution** | | | | |
| --- | --- | --- | --- | --- | --- | --- | --- |
| **Taxonomic level** | **mean** | **stdev** | **min** | **Lower Quartile** | **median** | **Upper Quartile** | **max** |
| **No hits_0-4 cm_** | 263 | 181 | 29 | 82 | 231 | 448 | 1907 |
| **No hits_10-15 cm_** | 232 | 175 | 29 | 73 | 164 | 403 | 1213 |
| **Not Assigned_0-4 cm_** | 444 | 94 | 50 | 406 | 473 | 507 | 800 |
| **Not Assigned_10-15cm_** | 445 | 101 | 50 | 410 | 480 | 511 | 739 |
| **Root_0-4 cm_** | 454 | 85 | 54 | 431 | 480 | 505 | 629 |
| **Root_10-15 cm_** | 457 | 90 | 44 | 429 | 485 | 512 | 876 |
| **Cellular_0-4 cm_** | 455 | 85 | 44 | 430 | 480 | 507 | 781 |
| **Cellular_10-15cm_** | 456 | 90 | 50 | 429 | 484 | 512 | 812 |
| **Kingdom_0-4 cm_** | 447 | 94 | 45 | 421 | 478 | 505 | 675 |
| **Kingdom_10-15cm_** | 451 | 96 | 50 | 423 | 483 | 511 | 639 |
| **Phylum_0-4 cm_** | 448 | 91 | 46 | 422 | 477 | 503 | 655 |
| **Phylum_10-15 cm_** | 447 | 91 | 46 | 422 | 477 | 503 | 655 |
| **Genus_0-4 cm_** | 451 | 90 | 51 | 425 | 479 | 507 | 668 |
| **Genus_10-15 cm_** | 455 | 91 | 57 | 431 | 483 | 510 | 613 |
| **Leaves_0-4 cm_** | 449 | 92 | 42 | 423 | 479 | 506 | 626 |
| **Leaves_10-15 cm_** | 446 | 100 | 42 | 415 | 481 | 510 | 1458 |
