# Supplementary material for: A metagenomic study of methanotrophic microorganisms in Coal Oil Point seep sediments
Source: BMC Microbiol. 2011 Oct 4;11:221. doi: 10.1186/1471-2180-11-221 (PMC3197505; doi:10.1186/1471-2180-11-221)
Supplement: Additional file 5 — Table S5. Genomes used for KAAS annotation. [file 1471-2180-11-221-S5.DOC]

**Table S5: Genomes used for KAAS annotation**

| **KAAS-code** | **species** | **Phyla/class** |
| --- | --- | --- |
| **aca** | *Acidobacterium capsulatum* | *Acidobacteria* |
| **sma** | *Streptomyces avermitilis* | *Actinobacteria* |
| **mag** | *Magnetospirillum magneticum* | *Alphaproteobacteria* |
| **bja** | *Bradyryhizobium japonicum* | *Alphaproteobacteria* |
| **swi** | *Sphingomonas wittichii* | *Alphaproteobacteria* |
| **aae** | *Aquifex aeolicus* | *Aquificae* |
| **fjo** | *Flavobacterium johnsoniae* | *Bacteroidetes* |
| **chu** | *Cytophaga tutchinsonii* | *Bacteroidetes* |
| **bam** | *Burkholderia cepacia* | *Betaproteobacteria* |
| **nmu** | *Nitrosospira multiformis* | *Betaproteobacteria* |
| **cyt** | *Cyanothece* sp. ATCC 51142 | *Canobacteria* |
| **cph** | *Chlorobium phaeobacteroides* DSM 266 | *Chlorobi* |
| **tpe** | *Thermofilum pendes* | *Crenarchaeota* |
| **dge** | *Deinococcus geothermalis* | *Deinococcus-Thermus* |
| **dde** | *Desulfovibrio desulfuricans* G20 | *Deltaproteobacteria* |
| **gur** | *Geobacter uraniumreducens* | *Deltaproteobacteria* |
| **dol** | *Desulfococcus oleovarans* | *Deltaproteobacteria* |
| **sfu** | *Syntrophobacter fumaroxidans* | *Deltaproteobacteria* |
| **dth** | *Dictyoglomus thermophilum* | *Dyctioglomi* |
| **emi** | *Elusimicrobium minutum* | *Elusimicrobia* |
| **sun** | *Sulfurovum* sp. NBC37-1 | *Epsilonproteobacteria* |
| **tdn** | *Sulfurimonas denitrificans* | *Epsilonproteobacteria* |
| **mac** | *Methanosarcina acetivorans* | *Euryarchaeota* |
| **afu** | *Archaeoglobus fulgidus* | *Euryarchaeota* |
| **mbu** | *Methanococcoides burtonii* | *Euryarchaeota* |
| **mem** | *Methanoculleus marsinigri* | *Euryarchaeota* |
| **rci** | Uncultured methanogenic archaeaon RC-1 | *Euryarchaeota* |
| **fsu** | *Fibrobacter succinogenes* | *Fibrobacteres* |
| **cth** | *Clostridium thermocellum* | *Firmicutes* |
| **fnu** | *Fusobacterium nucleatum* | *Fusobacteria* |
| **mca** | *Methylococcus capsulatus* | *Gammaproteobacteria* |
| **tcx** | *Thiomicrospira crunogena* | *Gammaproteobacteria* |
| **noc** | *Nitrosococcus oceani* | *Gammaproteobacteria* |
| **sse** | *Shewanella sediminis* | *Gammaproteobacteria* |
| **neq** | *Nanoarchaeum equitans* | *Nanoarchaeota* |
| **rba** | *Rhodopirellula baltica* | *Planctomycetes* |
| **tde** | *Treponema denticola* | *Spirochaetes* |
| **cau** | *Chloroflexus aurantiacus* | *Chloroflexi* |
| **nmr** | *Nitrosopumilus maritimus* | *Thaumarchaeota* |
| **tma** | *Thermotoga maritima* | *Thermotogae* |
